# Further Evidence That Female *Anoplophora glabripennis* (Coleoptera: Cerambycidae) Utilizes Photo-Degradation to Produce Volatiles That Are Attractive to Adult Males

**DOI:** 10.3390/insects15120923

**Published:** 2024-11-26

**Authors:** Damon Crook, Jacob Wickham, Lili Ren, Zhichun Xu, Tappey H. Jones, Melissa Warden, Allard Cossé

**Affiliations:** 1 Forest Pest Methods Laboratory, USDA-APHIS-PPQ-S&T, 1398 West Truck Road, Buzzards Bay, MA 02542, USA; 2 Institute of Zoology, Chinese Academy of Sciences, Beijing 100101, China; 3 Key Laboratory for Forest Pest Control, College for Forestry, Beijing Forestry University, Beijing 100101, China; 4 Department of Chemistry, Virginia Military Institute, Lexington, VA 24450, USA

**Keywords:** *Anoplophora glabripennis*, Asian longhorned beetle, cuticular hydrocarbons, oxidation, pheromone

## Abstract

The Asian longhorned beetle, *Anoplophora glabripennis* (Coleoptera: Cerambycidae: Lamiinae), is a serious pest of over 43 species of hardwood trees in North America, China and Europe. The development of an effective lure and trap for monitoring *A. glabripennis* has been hindered by the fact that mate finding involves a rather complex series of behaviors and responses to several chemical (and visual), cues. Adults (female-biased) locate a tree via host kairomones. Research has demonstrated that female contact pheromone components are precursors that undergo abiotic oxidation to yield attractive volatile components. Males also produce a pheromone to attract other adults nearby before the final step of recognition by males to the female-produced trail and contact pheromones. Our research aimed to identify new female-produced components from ozone and UV-treated *A. glabripennis* body washes and test them for behavioral activity using laboratory and field assays. The ozone and UV treatment of virgin female extract yielded sixteen aldehydes, nine of which were found in trace amounts. All sixteen aldehydes elicited antennal responses (GC-EAD) in both males and females, although responses were clearer and more distinct with standards of hexanal, heptanal, octanal, nonanal, decanal, undecanal and dodecanal. Olfactometer assays showed that males were highly attracted to a blend of these seven aldehydes. Females did not show any attraction to the blend in olfactometer assays. Despite low population levels during field tests in 2018 in China, traps containing the aldehyde blend detected *A. glabripennis* on a weekly basis over 6 weeks and caught significantly more adults (mainly males) than control ‘flight intercept panel’ traps. Field tests in China in 2019 that used the aldehyde blend along with a three-component host blend lure also caught significantly more males (nearly 5×) than blank control traps.

## Simple Summary

Recent research has demonstrated that contact pheromone components of the female Asian longhorned beetle, *Anoplophora glabripennis* (Coleoptera: Cerambycidae: Lamiinae), are potential precursors that can undergo abiotic oxidation to yield volatile attractants. That study uncovered the basic mechanism of female volatile production, but the identified component blend was incomplete. The main aim of this study was to identify the missing volatile components from female body wash extracts and test them for behavioral activity using laboratory assays and field trials. We identified several new components from ozone- and UV-treated female extracts using coupled gas chromatography–mass spectrometry (GC-MS) and gas chromatography–electroantennographic detection (GC-EAD). Male adult beetles were highly attracted to a blend of these seven new components in olfactometer tests, and males were primarily attracted to traps in the field when this seven-component blend was used. These newly identified compounds are discussed in relation to pheromones and host volatiles that *A. glabripennis* potentially utilizes in mate location.

## 1. Introduction

The Asian longhorned beetle, *Anoplophora glabripennis* (Motschulsky) (Coleoptera: Cerambycidae: Lamiinae), is a serious invasive pest species in several regions of the world [1]. In North America, *A. glabripennis* has caused the destruction of thousands of hardwood trees in New York (1996), Illinois (1998), New Jersey (2002), Massachusetts (2008) and Ohio (2011) [2,3,4,5,6,7]. The beetle has a broad host range, attacking over 43 species of hardwood trees such as maples, elms, ash, poplars and willows [8,9]. As of June 2020, South Carolina became the sixth US state to detect an infestation of *A. glabripennis*. Although infestations have been eradicated in New Jersey and Illinois, eradication efforts continue in New York, Massachusetts, Ohio and North Carolina [10]. Between 1998 and 2006, the Animal Plant Health Inspection Service (APHIS) assessed the costs of eradication measures in the US at $249 million [11]. Estimates of potential economic damage by *A. glabripennis*, if left unchecked, run into billions of US dollars for urban forests alone [12].

Eradication programs rely on the visual identification of the beetles and their damage to find infested trees and delimit *A. glabripennis* populations. Heavily infested trees can be found readily, but surveyors are much less efficient at spotting trees with minimal damage. Improved survey methods could greatly reduce program costs and/or enhance the ability to detect *A. glabripennis*, especially in areas away from the centers of infestations. Lures that have been developed to date, based on male-/female-produced pheromones or host plant volatiles (or a combination of the two), appear to be only weakly effective in field assays and/or contain components that are prohibitively expensive for use in large-scale monitoring programs. Male *A. glabripennis* produce two functionalized dialkyl ethers, 4-(*n*-heptyloxy)butanal and 4-(*n*-heptyloxy)butan-1-ol, that elicit GC-EAD responses in females [13] and are attractive to females in laboratory assays but show limited field attraction [13,14,15]. This moderate attraction in field trapping studies suggested that the male components may be ‘short range’ attractants or missing important additional unidentified components. A third, male-produced sesquiterpene component was identified in 2014 [16] as (3*E*,6*E*)-*α*-farnesene. When (3*E*,6*E*)-*α*-farnesene was combined with 4-(*n*-heptyloxy)butan-1-ol and 4-(*n*-heptyloxy)butanal, the attraction of both sexes in laboratory assays increased compared to 4-(*n*-heptyloxy)butan-1-ol and 4-(*n*-heptyloxy)butanal alone. Extensive field tests were not carried out on (3*E*,6*E*)-*α*-farnesene due to its lack of availability. Significant attraction to 4-(*n*-heptyloxy)butan-1-ol and 4-(*n*-heptyloxy)butanal has been observed in field assays when the components were combined with host kairomones such as (-)-linalool, (*Z*)-3-hexen-1-ol and *β*-caryophyllene [15,17,18]. A recent study [19] found a group of sesquiterpenes (*α*-longipinene, *α*-cubebene, *α*-ylangene, (-)-*α*-copaene, *α*-bergamotene, *β*-caryophyllene, and *α*-farnesene) in genital extracts from virgin females. These sesquiterpenes elicited antennal responses in males. In Y-tube behavioral assays significant attraction to *α*-longipinene was observed in male and female adults. Field tests showed limited (but significant) attraction to *α*-longipinene when combined with *α*-cubebene and *β*-caryophyllene [19]. The testing of these three antennally active sesquiterpenes likely led to an incomplete blend that resulted in reduced trap attraction. The *α*-longipinene used in that study was also a mixture of isomers (the actual attractive isomer remains unidentified at present). The presence of ‘non attractive’ isomers can weaken lure attraction [20,21] and may have also hindered insect attraction [19]. Sesquiterpene standards are notoriously difficult and expensive to synthesize, so their individual availability is very limited. The development of ‘economically viable’ lures for large scale monitoring programs has therefore relied on natural essential oils, such as manuka and phoebe oil, that contain naturally high concentrations of sesquiterpenes such as (-)-*α*-copaene and *α*-cubebene [22,23].

The development of an effective lure and trap for monitoring for *A. glabripennis* has also been rendered more complex because mate-finding and copulation involve a series of behaviors and responses to several chemical (and possibly visual) cues [1,16]. Up until 2012, species of the subfamily Cerambycinae were believed to have a three-step mating sequence [24]. Firstly, adults locate a tree via host kairomones. Secondly, males produce a pheromone to attract other adults nearby before the third step of recognition by males to female-produced trail and contact pheromones. In 2012, Wickham et al. [1] showed that there is a fourth step in the mating sequence of *A. glabripennis* where males respond to volatile, oxidized components of the female cuticular surface. Although gas chromatography–mass spectrometry (GC-MS) analysis has shown the long-chain hydrocarbon composition of cuticular waxes of male and female *A. glabripennis* to be very similar, Zhang et al. [25] showed that five monounsaturated compounds were consistently more abundant in female extracts. These compounds were identified as (*Z*)-9-tricosene, (*Z*)-9-pentacosene, (*Z*)-7-pentacosene, (*Z*)-9-heptacosene and (*Z*)-7-heptacosene. A synthetic mixture of these five compounds (applied to micro-centrifuge tubes) stimulated copulatory behavior in males upon antennal and palpal contact. These compounds appear to be precursors that can undergo abiotic oxidation to yield volatile aldehyde components [1]. Laboratory-based Y-tube olfactometer tests in that study showed that male *A. glabripennis* were significantly attracted to ozonized female extracts when tested against crude female extracts. Further laboratory behavioral assays and field tests showed that three of the identified aldehydes (heptanal, nonanal and hexadecanal) were attractive to adult males. The authors claimed to have found several other unique virgin female-produced unsaturated compounds which were not identified. The current virgin female ‘attractant’ blend produced by abiotic oxidation is therefore incomplete. Our main goal was to identify these missing, virgin female-produced components created by the photo degradation of *A. glabripennis* body washes and test them for behavioral activity in laboratory and field assays.

## 2. Materials & Methods

### 2.1. Source of Insects

All adult virgin *A. glabripennis* (between 3–4 weeks old) used in laboratory studies were reared at the Forest Pest Methods Laboratory Insect Containment Facility (Buzzards Bay, MA, USA) using similar protocols to those of Crook et al. [16]. For this study, we used adults that were primarily from a Chinese strain (15th generation). Insects were reared on an artificial diet until adulthood, when they were fed cut shoots of striped maple (*Acer pensylvanicum*) until needed. Adults were kept in glass half-gallon mason jars (Ball Ltd., Chicago, IL, USA) with 7.5 cm diameter metal screen lids. Twigs were held in water-filled 120 mL plastic cups (Dart Corporation, Holt, MI, USA) at the bottom of each jar. A coffee filter (8 to 12 cup size, Melitta^®^, Clearwater, FL, USA) was placed at the bottom of each jar to act as a substrate for insects and absorbent for spilled water. All insects were kept at 25 °C, approx. 60% relative humidity and 16:8 h L:D. The light system, situated 30 cm above the adult beetles, consisted of four T5 fluorescent lamps (Deep Blue Professional, City of Industry, CA, USA). Two were 39-W Solarmax T5 10,000 K daylight lamps, and two were 39-W Solarmax T5 Actinic 03 lamps that emitted a max blue phosphors peak at 420 nm. Automatic timers were set so that the actinic lamps turned on at 06:30 h and shut off at 2100 h. The daylight lamps turned on at 1030 h and turned off at 1530 h.

### 2.2. Oxidation and Analysis of Female Cuticular Hydrocarbon Extracts

Oxidation extracts were prepared using similar methods described by Wickham et al. [1]. Female extracts (*n* = 24 virgin females, 3–4 weeks old) were prepared by immersing individual live female beetles in 2 mL hexane (HPLC Grade, Fisher Chemicals, NJ, USA) in 22 mL glass vials (Supelco Inc., Bellefonte, PA, USA). Adults were chilled for 10 min in a standard −5 °C freezer before washing as this helped prevent insects from regurgitating internal fluids when dipped in hexane. Samples were gently shaken for 2 min and then concentrated to 0.5 mL by evaporation under nitrogen. The extracts were pooled before being oxidized. The extracts were oxidized by bubbling a slow stream of ozone through them at 0 °C for 5 min via an ozone generator (model L11, Pacific Ozone, Benicia, CA, USA) at a flow rate of <1 mL/min [1,26]. The samples were treated with one drop of dimethylsulfide and allowed to warm to room temperature. The volatile compounds derived from the ozonized female extracts were identified using coupled gas chromatography–mass spectrometry on a Shimadzu QP-2010 GC-MS equipped with an RTX-5, 30 m × 0.25 mm i.d. column. The instrument was programmed from 60 to 250 °C at 10°/min. Analysis by GC/MS showed the presence of aldehydes. To establish their precise structures, the solutions were treated with one drop (ca. 5 mg) of 1,1-dimethylhydrazine (Sigma Aldrich, St. Louis, MO, USA) [27]. The resulting dimethylhydrazones were apparent by the base peak at *m*/*z* = 86 and an intense parent ion in their mass spectrum. Aldehyde components (Table 1) were identified (using the same GC method) based on their mass spectra (NIST version 2.0, 2002) and the mass spectra of their dimethylhydrazones and by a comparison of retention indices and their mass spectra with available synthetic standards (Sigma Aldrich, St. Louis, MO, USA). Commercially unavailable aldehydes were prepared by the pyridinium chlorochromate oxidation of the corresponding commercially available alcohols [28]. Photo-oxidation extracts (UV treated female washes, n = 9) were prepared by placing female-washed hexane extracts into a capped Quartz High Precision Suprasil Cell (10 mm Light Path, Hellma Analytics, Plainview, NY, USA). Cell samples were then placed in a Forma Class II A2 Biological Safety Cabinet (Thermo Fisher Scientific, Waltham, MA, USA) under the UV light setting for 19 h followed by concentrating to 1 female equivalent (FE) per 10 µL of hexane.

### 2.3. Electrophysiological Analysis (GC/EAD)

The coupled GC/EAD system used was as previously described by Crook et al. [16] with a few modifications. Samples or aldehyde standards (2 μL, in hexane) were injected in splitless mode onto a Hewlett Packard (Agilent Technologies Inc. Santa Clara, CA, USA) 6890 gas chromatograph with a DB-5MS-DG column (30 m × 0.25 mm ID, 0.25 μm film thickness; J & W Scientific Inc., Folsom, CA, USA) and a 1:1 effluent splitter that allowed for the simultaneous FID and EAD detection of the separated volatile compounds. Helium was the carrier gas (2.5 mL/min). Oven temperature was held at 50 °C for 2 min, programmed to 280 °C at 10 °C/min and held for 15 min. Injector temperature was 275 °C. The GC outlets for the EAD and FID were 280 °C. The column outlet for the EAD was initially held in a water-cooled humidified air stream (20 °C) flowing at approximately 200 mL/min over the prepared antennae of adult *A. glabripennis* attached to an EAG probe (Syntech, Hilversum, The Netherlands). We suspected that aldehydes over 12 carbons long were condensing on the glass ‘jacket’ of the water- cooled airstream before reaching the antennal prep, so we replaced the water-cooled glass outlet with a digitally controlled effluent conditioner assembly (Type EC-03, Syntech, Hilversum, The Netherlands). The column outlet temperature was set at 300 °C. This latter setup was used to verify antennal activity for 16 aldehyde standards (approximately 500 ng per µL each).

Antennal recordings were prepared by cutting a single antenna at the base of the head of an adult beetle and removing the lower pedicel and scape. A size 1 insect pin (Bioquip Products, Compton, CA, USA) was used to make three holes on the first flagellomere as well as the third flagellomere from the tip. Holes were made deep enough to make a clean opening in the cuticular surface to allow conducting gel (Spectra 360, Parker Laboratories, Fairfield, NJ, USA) to form an uninterrupted connection to the EAG probe. One of the electrodes on the probe was extended with gold wire (20 mm long) to accommodate the long length of the antennal preparation (this was connected to the third flagellomere from the tip using conducting gel). This method preserved the tip of the antennae, eliminating the risk of removing vital sensillae specifically located there [29]. The EAG probe was connected to an IDAC-232 serial data acquisition controller (Syntech, Hilversum, The Netherlands). Signals were stored and analyzed on a PC equipped with the program EAD (version 2.6, Syntech, Hilversum, The Netherlands).

### 2.4. Olfactometer Bioassays of Oxidized Cuticular Extracts and Synthetic Aldehydes

All behavioral assays were conducted in a walk-in environmental chamber (25 °C, 60% RH). A Y-tube olfactometer (Analytical Research Systems Inc., Gainesville, FL, USA) was used to test the biological activity of potential attractants. The glass Y-tube (3.5 cm internal diameter) had a 15 cm main stem that branched into two 13 cm arms angled at 90°. Each arm was then connected to a separate glass tube that contained the stimulus or a solvent/blank control. Charcoal-filtered air was bubbled through distilled water and then into each of the two arms at 1.0 L/min using a 2-channel air delivery system (Analytical Research Systems Inc., Gainesville, FL, USA). The Y-tube was held at a 15° angle upward from horizontal on a custom-built holder placed 0.5 m below the light source. As cerambycid beetles have been reported to be sedentary or agitated under laboratory light conditions [30], we used a lighting system that closely approximated the wavelengths and intensity of natural light. The lights used consisted of four ‘T5’ fluorescent lamps (Deep Blue Professional, City of Industry, CA, USA). Two lamps were 39 W Solarmax T5 10,000K daylight lamps, and two were 39 W Solarmax T5 Actinic lamps. The lights above the olfactometer arena had an output of approximately 450 lx. Male and female virgin adults between 15 and 40 days old were used for all olfactometer bioassays. Insects were fed on twigs until used in tests, i.e., with no starvation period. A total of 25 replicates were completed for each treatment, using one beetle per replicate. Adults were not re-used for olfactometer assays on the day of testing.

The first set of olfactometer bioassays (Table 2) was aimed at determining male and female response to a mix of seven aldehydes (based on GC-EAD results). Five stimulus treatments were prepared for the first set of olfactometer bioassays. The first treatment was a mix of the first seven antennally active aldehydes, produced and identified after the ozone treatment of female body washes (7 × ALD). This consisted of hexanal (6:ALD), heptanal (7:ALD), octanal (8:ALD), nonanal (9:ALD), decanal (10:ALD), undecanal (11:ALD) and dodecanal (12:ALD). Two concentrations were prepared of the 7 × ALD mix. One had 1 µg of each aldehyde per 10 µL of hexane. The second had 10 µg of each aldehyde per 10 µL of hexane. Both treatments had an approximate ratio of 1:1:1:1:1:1:1. The third treatment (7 × ALD Fem Ratio) was a mix of the seven aldehydes described above but at the approximate ratio observed on the GC-MS after ozone treatment (1:3:1:15.5:1:1:1; with 1 part = 1 µg). The fourth treatment consisted of six virgin female hexane body washes that had been UV-treated overnight and concentrated down to one female equivalent (FE) per 10 µL of hexane (1FE Body wash UV treated).

These four treatments were dispensed onto a strip of filter paper (10 × 40 mm) and placed in the tube connected to one arm of the olfactometer. An identical filter paper strip with the same amount of hexane was placed in the other arm of the olfactometer. The fifth treatment used a 0.25 mL polyethylene tube (CAT # 02-681-449, Fisherbrand, Pittsburgh, PA, USA) as a release device. Mineral oil (200 mg) was loaded into a tube along with 1 mg of each of the seven aldehydes (each dissolved in 10 µL hexane) as described previously (the control was a tube loaded with 200 mg of mineral oil and 70 µL of hexane). Mineral oil-filled tubes (with closed caps) were left to age in a fume hood for 24 h before being used.

The second set of olfactometer bioassays (Table 3) aimed to determine male behavioral responses to seven-component aldehyde blends (7 × ALD) as well as two concentrations of a three-component ‘host’ blend (currently used in *A. glabripennis* surveys). The two 7 × ALD concentrations (described above) were tested along with a seven-component aldehyde blend that consisted of the most abundant aldehydes observed after the ozone treatment of body washed females (termed ‘non-trace’ 7 × ALD blend). These were heptanal (7:ALD), nonanal (9:ALD), tetradecanal (14:ALD), hexadecanal (16:ALD), heptadecanal (17:ALD), octadecanal (18:ALD) and eicosanal (20:ALD). The non-trace 7 × ALD blend consisted of 10 µg of each aldehyde in 10 µL of hexane. The three-host blends consisted of (-) linalool, (*Z*)-3-hexen-1-ol and trans-caryophyllene (1 µg and 10 µg per 10 µL of hexane, each at a ratio of 1:1:1).

The Y-tube was rinsed with acetone between each individual test. Treatment and control arms were alternated every other replicate to avoid possible positional effects. For each test, a single male or female beetle was placed at the end of the main stem and given 5 min to choose between the two stimuli. A choice was recorded when the beetle passed a line, 8 cm beyond the branch point of each arm. No choice was recorded if the beetle failed to pass either line after the 5 min period. Treated filter paper strips were replaced with new ones after 45 min.

Insects that did not make a choice were excluded from statistical analysis. All experiments were conducted between 1100 and 1500 h, when beetles appeared to be most active. To assess whether the test stimulus attracted more beetles than the solvent control in Y-tube olfactometer bioassays, an χ^2^ goodness-of-fit test was performed. Values of χ^2^ > 3.84 with 1 d.f. were significant at α = 0.05 (Epistat 2.1, Austin, TX, USA).

### 2.5. Field Responses of A. glabripennis to Potential Attractants

We evaluated the attractiveness of suspected ‘female based’ attractants in randomized complete block field experiments using flight intercept panel traps (Chemtica Internacional, S.A., Heredia, Costa Rica) from June through August of 2018 and 2019. The top cone of each trap was removed and then attached to a main tree bole at head height (~1.8 m) using three 48″ jumbo nylon cable ties (ULINE, Pleasant Prairie, WI, USA). Collection cups were filled with saline solution. Lures were attached to the opening in the traps’ center. Traps were placed 15 to 20 m apart.

In 2018, two seven-component aldehyde lure treatments (high and low release) were tested against blank control traps. The first high release lure treatment consisted of three bubble cap devices (Synergy Semiochemicals, Delta, BC, Canada) each releasing approximately 30 mg per day. The first bubble cap was loaded with hexanal (C6:ALD) and heptanal (C7:ALD). The second was filled with octanal (8:ALD) and nonanal (9:ALD). The third contained decanal (10:ALD), undecanal (11:ALD) and dodecanal (12:ALD). The second low release lure treatment consisted of the same three bubble caps but with loadings that were 10× less concentrated (approximately 3 mg per day release rate). These 10% loadings were diluted with acetyl tributyl citrate (a low volatile ester).

Hexanal- and heptanal-loaded bubble caps for both high and low release treatments were replaced every 2 weeks due to their high volatility. The 2018 field test was set up within the Yongle Ecological Park (39°37′32.01″ N, 116°46′20.94″ E, elev. 16 m) and Grand Canal area of Beijing, China (39°51′53.67″ N, 116°44′20.44″ E, elev. 21 m). This park contained willow trees that were known to have a low-level population of *A. glabripennis*. Twenty replicates were set out on 29 June 2018 and checked weekly until 12 August 2018. Lures were re-randomized each week when traps were checked for captured beetles.

In 2019, a field experiment was set up on 12 July in the city of Jiuquan in Gansu, China (poplar grove: 39°47′11.25″ N, 98°22′6.78″ E, elev 1561 m; Jinyu Park: 39°44′36.23″ N, 98°26′38.40″ E, elev. 1568 m). This experiment had 5 treatments: 1. seven-aldehyde blend (30 mg day); 2. seven-aldehyde blend (30 mg day) plus a three-component kairomone lure (60 mg day) (47.5% racemic linalool, 10.5% (*Z*)-3-hexen-1-ol and 42% trans-caryophyllene); 3. The three-component kairomone lure; 4. Male-produced two-component pheromone (4-(*n*-heptyloxy)butan-1-ol and 4-(*n*-heptyloxy) butanal, 1 mg per day each) plus the three-component kairomone lure; 5. Unbaited control.

Treatments were replicated fifteen times and checked weekly from 19 July to 24 August. The first six replicates were set up in an area populated by poplar trees. Replicates 7 to 15 were set up in an area mainly populated by willow trees.

Mean trap catches were analyzed by fitting Poisson generalized linear models in R statistical computing software [31]. The trap catch over the five-week period was the response variable, the treatment was the predictor variable, and a fixed effect was used for the block. For the 2019 experiment, the sex of captured beetles was also a predictor, and an interaction between sex and treatment was tested. A random effect was used for the trap (with two observations from each trap: female counts and male counts), and the blocking factor was the study site. If the data showed “separation” [32] (e.g., where one treatment had 0 catch for all traps), then model fitting was carried out with Firth’s bias correction using the “brglm2” package [33]. Contrasts between treatment groups were constructed with the “emmeans” package [34], using a simulation technique from the multivariate t-distribution to adjust *p*-values for multiple comparisons.

## 3. Results

### 3.1. Oxidation and Analysis of Female Cuticular Hydrocarbon Extracts

**Figure 1:**
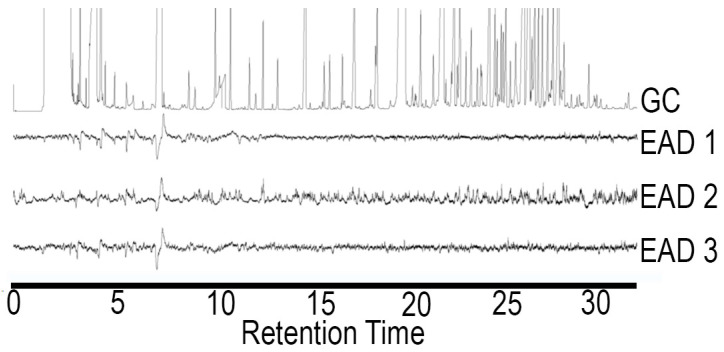
Typical EAD responses of three male *A. glabripennis* antennae to an ozone-treated female body wash sample.

**Figure 2:**
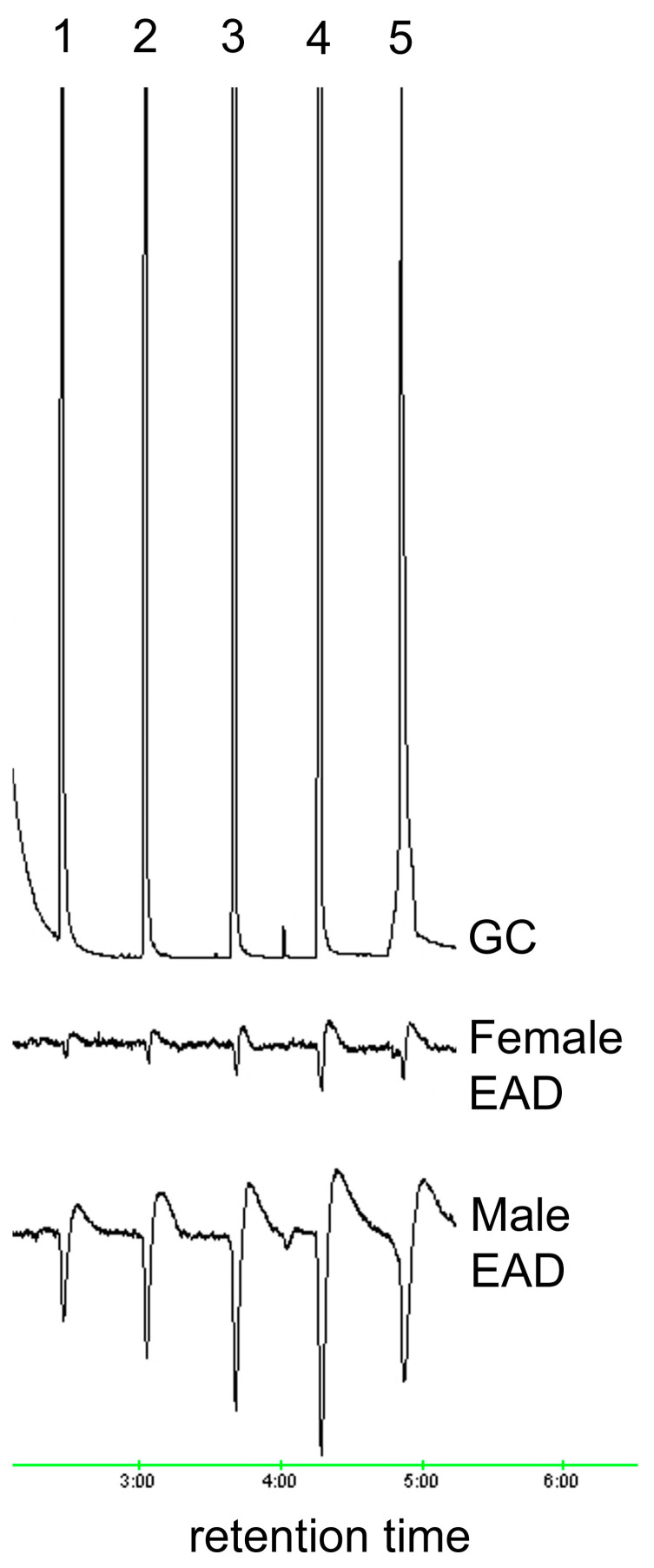
EAD responses of male and female *A. glabripennis* antennae to standards of (1) hexanal, (2) heptanal, (3) octanal, (4) nonanal and (5) decanal.

**Figure 3:**
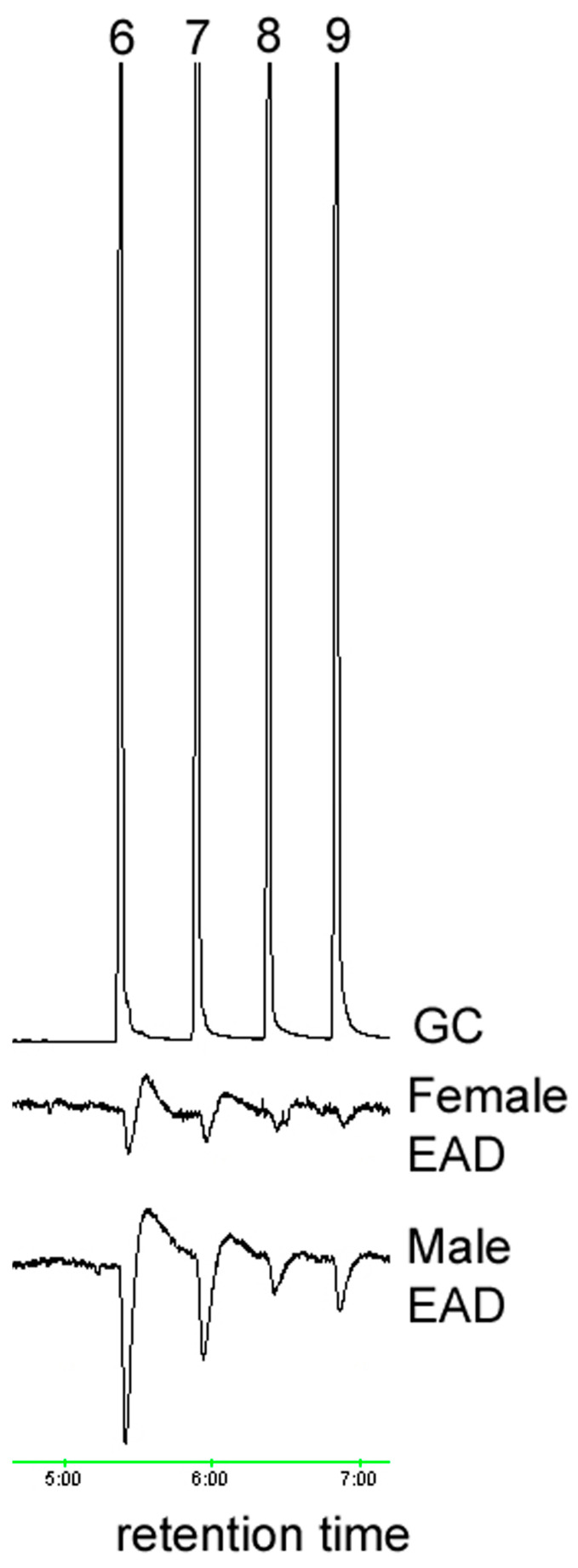
EAD responses of male and female *A. glabripennis* antennae to standards of (6) undecanal, (7) dodecanal, (8) tridecanal and (9) tetradecanal.

**Figure 4:**
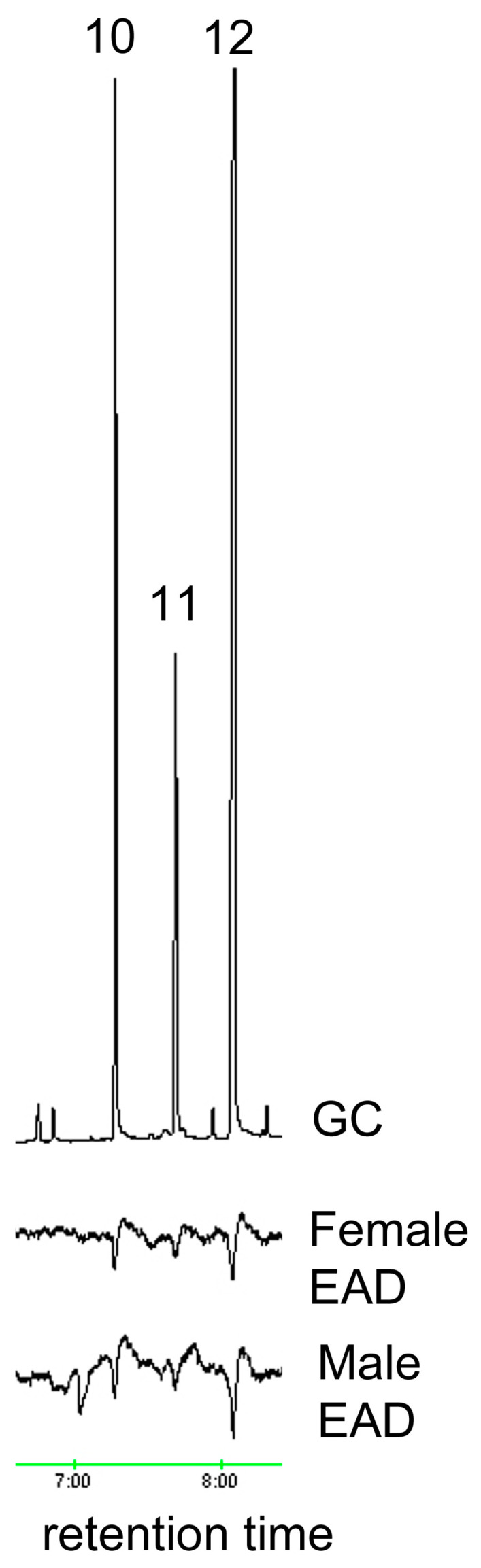
EAD responses of male and female *A. glabripennis* antennae to standards of (10) pentadecanal, (11) hexadecanal and (12) heptadecanal.

**Figure 5:**
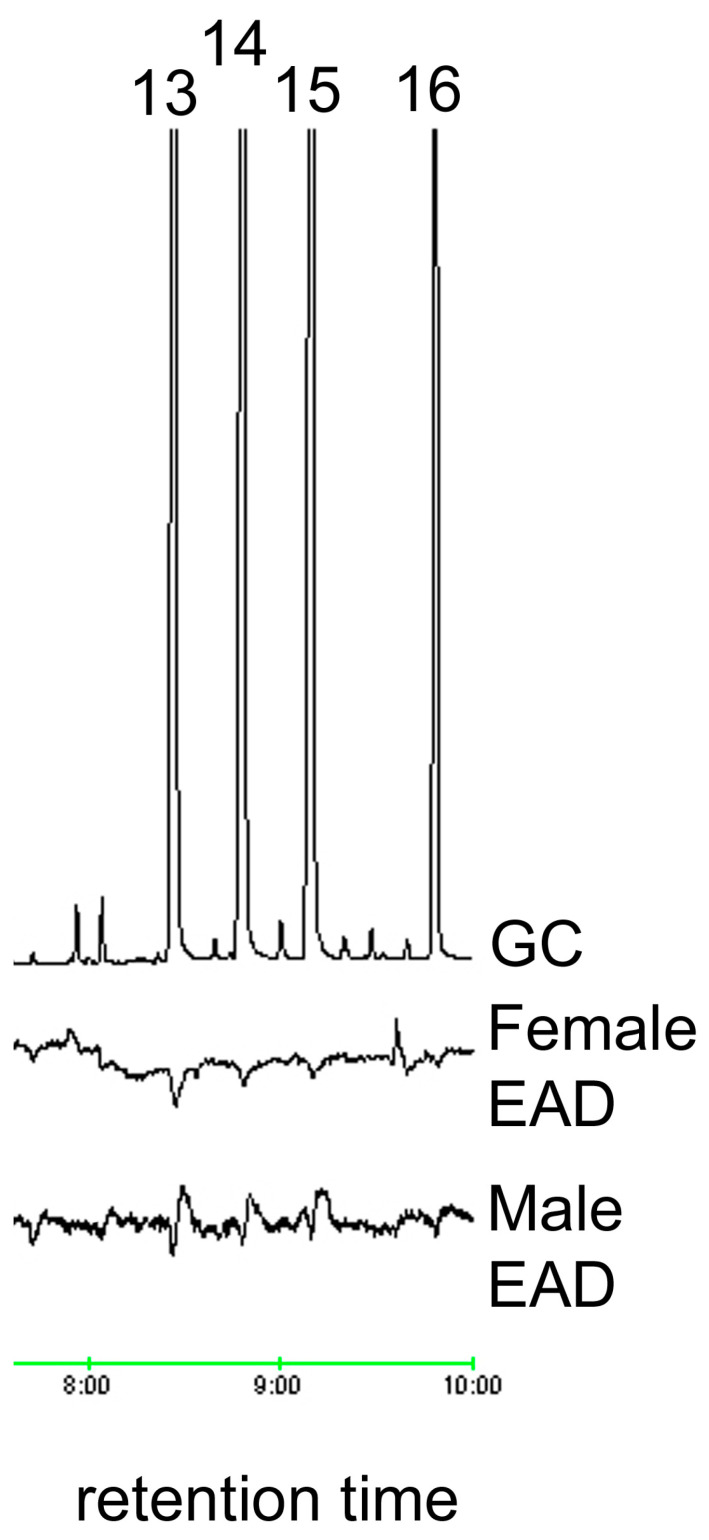
EAD responses of male and female *A. glabripennis* antennae to standards of (13) octadecanal, (14) nonadecanal, (15) eicosanal and (16) docosanal.

The ozone treatment of virgin female hexane washes yielded sixteen aldehydes, nine of which were found in trace amounts (Table 1). The seven most abundant products (7:ALD, 9:ALD, 14:ALD, 16:ALD, 17:ALD, 18:ALD and 20:ALD) were in the approximate ratio of 3:15.5:2.3:1.5:1.4:15.1:4.4. These seven most abundant products were obtained in a similar ratio for female washes that had been UV treated. All sixteen aldehyde components elicited antennal responses in both male and female *A. glabripennis* (Figure 1, Figure 2, Figure 3, Figure 4 and Figure 5). Antennal responses were clearer and more distinct with standards of 6:ALD, 7:ALD, 8:ALD, 9:ALD, 10:ALD, 11:ALD and 12:ALD.

### 3.2. Olfactometer Bioassays of Oxidized Cuticular Extracts and Synthetic Aldehydes

In Y-tube olfactometer bioassays, UV-treated virgin female cuticular body wash was significantly more attractive to adult male *A. glabripennis* than hexane controls (Table 2). The 7 × ALD blend was significantly more attractive to males than hexane controls at two different doses as well as when presented in a polyethylene tube in 200 µL of mineral oil. The 7 × ALD Fem Ratio treatment was also significantly more attractive to males than hexane controls. The 7 × ALD blend was not significantly attractive to adult female *A. glabripennis* at either 1 or 10 µg doses when compared to hexane controls (Table 2).

The three-component host blend (1 µg) was not attractive to male *A. glabripennis* over a hexane control (Table 3). At the 1 µg dose, male *A. glabripennis* were not significantly attracted to 7 × ALD + three-component host blend when the three-component host blend was offered as a control choice. Males were shown to significantly prefer the 7 × ALD + three-component host blend over the three-component host blend alone when it was offered at a dose of 10 µg. The ‘non-trace’ 7 × ALD blend was not attractive to male *A. glabripennis* by itself or in combination with the three-component host blend.

### 3.3. Field Responses of A. glabripennis to Potential Attractants

**Figure 6:**
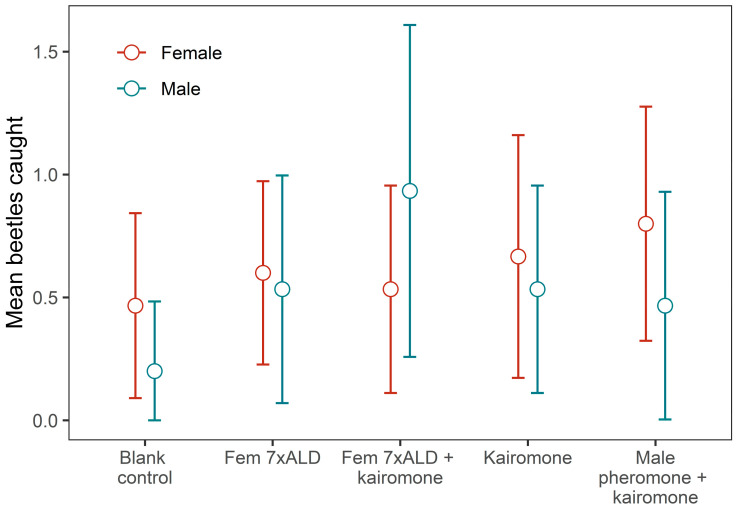
Observed mean trap catches of female and male *A. glabripennis* in 2019 field experiment. Bars are 95% CI for the mean. Kairomone = 47.5% racemic linalool, 10.5% (Z)-3-hexen-1-ol and 42% trans-caryophyllene (60 mg day). Pheromone = 4-(*n*-heptyloxy)butan-1-ol and 4-(*n*-heptyloxy)butanal (1 mg per day each).

The 2018 experiment captured nine *A. glabripennis*, seven at the high dose, two at the low dose and zero at the blank control. Dose had an overall effect on trap count (χ^2^ = 8.25, df = 2, *p* = 0.016). The high dose captured significantly more beetles than the blank control (*z* = 2.56, *p* = 0.03), whereas the low dose did not (*z* = 1.43, *p* = 0.31). The 2019 experiment captured 86 *A. glabripennis*, 46 females and 40 males, with mean catch per trap approximately 0.5–0.8 beetles for females and 0.2–0.9 beetles for males (Figure 6). The female 7 × ALD blend plus three-component kairomone lure caught more males than the other lures, but no lure was significantly preferred over the others, for either males or females ($\chi^{2}$ for interaction effect = 4.5, df = 4, *p* = 0.34). A reduced model with no interaction showed no differences in mean trap catch across lure treatments ($\chi^{2}$ = 5.0, df = 4, *p* = 0.29). Planned contrasts of the female 7 × ALD blend plus three-component kairomone lure against the blank control and against the female 7 × ALD lure on its own indicated that the combined female/kairomone lure was marginally significant over the blank control but not over the female 7 × ALD blend (Table 4). If we modeled male trap catch only, the combined female/kairomone lure had a higher catch than the blank control but not higher than the female 7 × ALD blend (Table 5).

## 4. Discussion

The results support and expand the findings by Wickham et al. [1] in that female *A. glabripennis* contact pheromone components are precursors that undergo photo degradation to yield other, attractive, volatile components. Wickham et al. [1] found that the ozone treatment of mated or virgin female solvent washes yielded six functionalized products (7:ALD, 9:ALD, 14:ALD, 16:ALD, 18:ALD and 20:ALD). The ratio of these products was observed to be slightly different depending on the mating status. Mated female washes produced an approximate ratio of 3:11:1:2:10:1. Virgin female washes produced a ratio of 3:22:1.5:3:10:1. From the six aldehydes Wickham et al. [1] reported that 7:ALD, 9:ALD and 16:ALD appeared to elicit antennal responses from males. Field tests in China showed that synthetic lures of these three aldehydes (in a ratio similar to that of virgin female extracts) caught more beetles than controls. Combinations of those aldehydes with (−)-linalool, linalool oxide, (*Z*)-3-hexen-1-ol, camphene, 3-carene and *β*-caryophyllene also caught more insects than controls and captured significantly more males. This provided the first field evidence of a female volatile ‘attractant’ for *A. glabripennis* and uncovered the basic mechanism of its production.

Our results show that ozone- and UV-treated virgin female body washes produced very similar results to Wickham et al. [1]. We also did not observe detectable levels of aldehyde components in extracts from chilled, live beetles that had not been oxidized or UV-treated. Wickham et al. [1] also found that photo-oxidations of female extracts achieved by exposure to sunlight (on glass microscope slides) yielded antennally active aldehydes in the same approximate ratios as those produced by an ozone treatment. We found seven functionalized products to be the most abundant components of our ozone-treated body washes. We found the same six aldehydes as Wickham et al. [1] as well as heptadecanal (17:ALD). We also detected nine other aldehyde components in trace amounts. Our results show that we were able to obtain clear antennal responses for all sixteen of these aldehydes, for both male and female adults. Wickham et al. [1] did not obtain detectable GC-EAD responses to tetradecanal (14:ALD), octadecanal (18:ALD) or eicosanal (20:ALD). Our differing results could be due to the use of slightly different electrophysiological antennal preparations. Wickham et al. [1] also reported barely detectable levels for the three aldehydes stated above, which would have made antennal detection more difficult to clarify. Our study showed that antennal responses (delivered by GC-EAD to the antennae at the same dose) were larger and more obvious for aldehydes with 12 carbons or fewer (6:ALD, 7:ALD, 8:ALD, 9:ALD, 10:ALD, 11:ALD and 12:ALD). We therefore focused our laboratory assays and field tests on those seven components. In olfactometer bioassays, males were equally attracted to those aldehydes either in an equal 1:1:1:1:1:1:1 ratio or at a ratio based on GC-MS analysis after ozone treatment. A blend of the most abundant seven aldehydes (7:ALD, 9:ALD, 14:ALD, 16:ALD, 17:ALD, 18:ALD and 20:ALD) produced after ozone treatment did not prove to be more attractive to males in laboratory assays. Male *A. glabripennis* therefore, tended to show greater attraction to the smaller, more volatile range of aldehydes produced through photo degradation.

In the last thirteen years, several studies have shown that host plant volatiles can synergize the attraction of cerambycid species to the pheromones they produce [35]. In the subfamily *Lamiinae*, several *Monochamus* species are more attracted to combinations of monochamol with α-pinene and ethanol than to monochamol itself [36,37,38,39]. Field trapping results have been mixed for *A. glabripennis*, with lures being relatively weak in terms of attraction [1,18,40,41]. Despite low population levels during field tests in 2018 in China, traps containing the aldehyde blend detected *A. glabripennis* on a weekly basis over 6 weeks and caught significantly more adults (mainly males) than control flight intercept traps. It is important to emphasize that in the context of surveillance for *A. glabripennis* within the United States, the detection of a single beetle is important so that delimitation and eradication measures can be planned. Our lab olfactometer bioassays and 2019 field assay using the three-component host blend showed mixed results. In olfactometer assays, male insects were significantly attracted to the 7 × ALD + three-component host blend over the three-component host blend by itself at the 10 µg loading but not at the lower 1 µg dose. The ‘non-trace’ 7 × ALD blend did not appear to display a synergistic interaction with the three-component host blend at the 10 µg dose. In our 2019 field test, we caught a total of 86 beetles over a 5-week period. Contrast tests showed that the addition of a three-component kairomone lure to the female 7 × ALD pheromone lure did not significantly improve trap catch over the female 7 × ALD pheromone lure by itself. The combined female/kairomone lure did, however, catch significantly more insects than the blank control traps. The female 7 × ALD pheromone and three-component kairomone lure also caught significantly more males than the blank control traps (nearly 5× more). This supports similar field experiments by Wickham et al. [1] where a marginal improvement in trap catch was observed when kairomone compounds were included in lure tests with the female-produced aldehyde components. Some aldehydes are abundant in nature, and some of the oxidation products reported here (heptanal and nonanal) are known kairomone enhancers of pheromones, when they are released from a host after feeding has taken place [1,42]. Smith et al. [43,44] observed that the female:male ratio of *A. glabripennis* arriving at uninfested *Acer mono* Maxim. trees shifted from 3.5:1 to 2:1 after females had begun feeding in the tree. Volatiles released from female feeding sites has also been suggested as a key factor for how male *Anoplophora malasiaca* (Thomson) locate mates [45,46]. Further investigations are needed to better understand how volatiles released from host tree feeding sites interact with female-produced volatiles. It is important also to note that the cuticular hydrocarbon composition (as well as the ratio of them) could vary with insect age, sex and mating status [1]. These variables should be examined further to better understand the complex mechanism of female volatile production in *A. glabripennis*.

Our GC-EAD results show that male and female *A. glabripennis* antennae respond to sixteen aldehydes produced by the oxidation of female cuticular components. Single-sensillum recordings may help elucidate the importance of these aldehydes in *A. glabripennis* mate location. A study by Wei et al. [47] performed single-sensillum recordings on male and female antennae of *A. glabripennis* using male-produced aggregation pheromone components in addition to some other odorants. They found that trichoid sensilla on the most distal antennal flagellomeres had olfactory sensory neurons (OSNs) with high-amplitude action potentials that were tuned to the male aldehyde (4-(*n*-heptyloxy)butanal) and alcohol (4-(*n*-heptyloxy)butan-1-ol) pheromone components. These trichoid sensilla did not respond to various plant volatiles that were tested. In other more proximally located sensilla, the authors found OSNs in blunt tipped basiconic sensilla that responded to plant related volatiles (especially terpenoids) such as (*E*,*E*)-alpha farnesene, (*E*)-*β*-farnesene, *β*-caryophyllene and eugenol. Further single-sensillum recordings of OSNs on the antennae of male and female *A. glabripennis* may help clarify the sensory neurophysiological importance of female produced attractants and help determine which of these photo-oxidation and/or degradation products is the most important in terms of potential lure development.

The conversion of relatively non-volatile cuticular lipids or hydrocarbons into volatile pheromones has been reported for several insect taxa [48], including Hemiptera [49,50], Hymenoptera [51,52,53], Coleoptera [1] and Diptera [54]. Zhang et al. [25] reported that the female-produced contact pheromone for *A. glabripennis* contained five olefins that elicited copulatory behavior in males. These were (*Z*)-9-tricosene, (*Z*) 9 pentacosene, (*Z*)-7-pentacosene, (*Z*)-9-heptacosene and (*Z*)-7-heptacosene. Three of these, (*Z*)-9-tricosene, (*Z*) 9-pentacosene and (*Z*)-9-heptacosene, are also cuticular components of the sawfly *Cephus cinctus* Norton (Hymenoptera: Cephidae) [50,55]. For *C. cinctus* these three components were shown to oxidize and produce volatiles that had pheromonal activity. Three other species of Hymenoptera, the yellow-headed spruce sawfly, *Pikonema alaskensis* Rohwer (Tenthredinidae) [56], *Macrocentrus grandii* Goidanich (Braconidae) [57] and the pine false webworm, *Acantholyda erythrocephala* (L.) (Pamphiliidae) [53], have all been shown to form volatile pheromone compounds from the abiotic oxidation of unbranched alkenes and alkenediol acetates.

The suite of mate-finding behaviors for *A. glabripennis* is further complicated by the fact that (*Z*)-9-tricosene, (*Z*)-9-pentacosene and (*Z*)-7-pentacosene (along with 2-methyldocosane) have also been identified as the four main components of a female-produced trail pheromone [58]. Assays showed that these four components allowed males to follow the trails of females on the substrate they walk on. Upon contact with the full four-component trail pheromone blend, a high percentage of males (87.5%) became ‘arrested’ and displayed a ‘pheromone releasing behavior’ previously described by Lacey et al. [59]. Their production for ‘peak male attraction’ appears to coincide with peak female sexual maturation. Hoover et al. [58] also showed that females appeared to be repelled by the four-component trail pheromone, indicating that it may function as an anti-aggregation (spacing) pheromone for females. It is important to note that virgin male *A. glabripennis* were highly responsive only to the two major trail pheromone components, 2-methyldocosane and (*Z*)-9-tricosene. In further studies, Graves et al. [60] evaluated the ability of virgin male *A. glabripennis* to follow the female trail pheromone after the removal of the terminal four antennal segments and/or the maxillary and labial palps. They found that males lacking palps were unable to respond behaviorally to the trail pheromone. Direct palpal contact, therefore, appeared to be necessary for males to be able to efficiently follow the trail pheromone, although the degree of volatilization by the pheromone components was not examined in that study.

(*Z*)-9-tricosene is therefore an important component of both the trail and contact pheromones of female *A. glabripennis* and is capable of oxidizing into further volatile attractive components. It is reported as having pheromonal activity for several different insect species but (*Z*)-9-tricosene (commonly known as muscalure) is mainly known for its role as the main mating attractant for the house fly, *Musca domestica* [61]. A recent study by Lima et al. [62] showed that (*Z*)-9-tricosene also acted as a potential sex pheromone for the invasive spotted wing fly, *Drosophila suzukii* (Matsumura) (Diptera: Drosophilidae).

Although we do not yet know how long the trail pheromone persists on the tree before it dissipates, our results indicate it is highly feasible that components such as (*Z*)-9-tricosene would eventually oxidize to volatile aldehydes that could attract male *A. glabripennis* over a longer distance to an area recently visited by adult females. The oxidation scheme and expected products from (*Z*)-9-tricosene are summarized by Bartelt et al. [51].

The abiotic oxidation of cuticular hydrocarbon components is a complex topic because it can be initiated by several factors such as chemical initiators, ultraviolet light and temperature [63]. These factors would favor the oxidation of cuticular components for *A. glabripennis* adults, which is diurnal and active in the crown of trees on sunny days. Oxidation rates could also potentially be enhanced or controlled by insect behavior such as wing fanning and grooming, which could possibly redistribute pheromonal precursors over the cuticle. The complex oxidation scheme and expected products from (*Z*)-9-tricosene are well-studied and are summarized by Bartelt et al. [51]. Further research should examine, identify and compare the volatile products of 2-methyldocosane and (*Z*)-9-tricosene with respect to their potential as attractants for *A. glabripennis*.

Mate location in *A. glabripennis* is clearly a complex system involving male- and female-produced pheromones along with several host kairomones. Our identification of several more ‘short range’ attractants support the theory that for mate location, adult *A. glabripennis* utilizes a series of events that are mediated by several relatively weak or short-ranged stimuli. Recent research by Xu et al. [19] suggests that α-longipinene, isolated from female ovipositors, is as attractive at high doses or when combined with hexadecanal or host kairomones. Future research should test whether α-longipinene enhances trap catch when used with additional potential synergists, such as the female produced attractants identified in this study. This will hopefully lead to a more effective and practical monitoring tool that will benefit survey and pest management programs for this important invasive beetle.

## Figures and Tables

**Table 1:** Aldehyde oxidation products of ozone treated female *A. glabripennis* cuticular components (t = trace).

Aldehyde	Retention Time (Min)	Relative Peak Area	Percent Ratio of Total Aldehydes
Hexanal	9.08	t	
Heptanal	9.98	3.0	6.9
Octanal	10.78	t	
Nonanal	11.51	15.5	35.9
Decanal	12.20	t	
Undecanal	12.80	t	
Dodecanal	13.45	t	
Tridecanal	14.02	t	
Tetradecanal	14.58	2.3	5.3
Pentadecanal	15.10	t	
Hexadecanal	15.60	1.5	3.5
Heptadecanal	16.20	1.4	3.2
Octadecanal	16.87	15.1	34.9
Nonadecanal	17.62	t	
Eicosanal	18.52	4.4	10.2
Docosanal	20.90	t	

**Table 2:** Male and female *A. glabripennis* responses to female extract (UV treated) and a seven-component aldehyde blend in an olfactometer. 7 × ALD correspond to hexanal, heptanal, octanal, nonanal, decanal, undecanal and dodecanal.

Sex	Odor Source 1	Odor Source 2	Choice 1	Choice 2	% Response to Choice 1	χ^2^	*p*
Male	7 × ALD (10 µg)	hexane (10 µL)	20	5	80	7.84	0.003
Male	7 × ALD (1 µg)	hexane (1 µL)	19	6	76	5.76	0.001
Male	7 × ALD (1 mg) in 200 µL Min Oil	Min Oil & Hexane	19	6	76	5.76	0.001
Male	1FE Body Wash UV treated	10 µL hexane (10 µL)	22	3	88	12.96	0.001
Male	7 × ALD Fem Ratio (10 µg)	hexane (10 µL)	21	4	84	10.24	0.001
Female	7 × ALD (10 µg)	hexane (10 µL)	8	17	32	2.56	NS
Female	7 × ALD (1 µg)	hexane (1 µL)	12	13	48	0	NS

**Table 3:** Male and female *A. glabripennis* responses to seven-component aldehyde blends and a three-component host volatile blend in an olfactometer.

Sex	Odor Source 1	Odor Source 2	Choice 1	Choice 2	% Response to Choice 1	χ^2^	*p*
Male	3 Host Blend (1 µg)	hexane (10 µL)	11	14	44	0.16	NS
Male	7 × ALD (1 µg) + 3 Host Blend (1 µg)	hexane (20 µL)	20	5	80	7.84	0.003
Male	7 × ALD (1 µg) + 3 Host Blend (1 µg)	3 Host Blend (1 µg)	12	13	48	0	NS
Male	7 × ALD (10 µg) + 3 Host Blend (10 µg)	3 Host Blend (10 µg)	19	6	76	5.76	0.001
Male	Non Trace 7 × ALD (10 µg) + 3 Host Blend (10 µg)	3 Host Blend (10 µg)	15	10	60	0.64	NS
Male	Non Trace 7 × ALD (10 µg)	Hexane (10 µL)	15	10	60	0.64	NS

**Table 4:** Contrasts of total *A. glabripennis* catch rates for 2019 field experiment, in terms of rate ratios and their standard errors (SE).

Contrast	Rate Ratio	SE	*z*	*p*
Fem 7 × ALD/blank control	1.72	0.72	1.30	0.28
Fem 7 × ALD + kairomone/blank control	2.21	0.89	1.98	0.06
Fem 7 × ALD + kairomone/Fem 7 × ALD	1.29	0.45	0.74	0.47

**Table 5:** Contrasts of male *A. glabripennis* catch rates for 2019 field experiment, in terms of rate ratios and their standard errors (SE).

Contrast	Rate Ratio	SE	*z*	*p*
Fem 7 × ALD/blank control	2.67	1.81	1.45	0.17
Fem 7 × ALD + kairomone/blank control	4.67	2.97	2.42	0.02
Fem 7 × ALD + kairomone/Fem 7 × ALD	1.75	0.78	1.26	0.23

## Data Availability

Data are available upon request.

## References

[1] Wickham J.D., Xu Z., Teale S.A. (2012). Evidence for a female-produced, long range pheromone of *Anoplophora glabripennis* (Coleoptera: Cerambycidae). Insect Sci..

[2] Cavey J.F., Hoebeke E.R., Passoa S., Lingafelter S.W. (1998). A new exotic threat to North American hardwood forests: An Asian longhorned beetle, *Anoplophora glabripennis* (Motschulsky) (Coleoptera: Cerambycidae) I. Larval description and diagnosis. Proc. Entomol. Soc. Wash..

[3] Poland T.M., Haack R.A., Petrice T.R. (1998). Chicago joins New York in battle with Asian longhorned beetle. Newsl. Mich. Entomol. Soc..

[4] Haack R.A., Shaw K., Mastro V.C., Ossenbruggen S., Raimo B.J. (1997). New York’s battle with the Asian longhorned beetle. J. For..

[5] Haack R.A. (2006). Exotic bark- and wood-boring Coleoptera in the United States: Recent establishments and interceptions. Can. J. For. Res..

[6] Haack R.A., Herard F., Sun J.H., Turgeon J.J. (2010). Managing invasive populations of Asian longhorned beetle and citrus longhorned beetle: A worldwide perspective. Annu. Rev. Entomol..

[7] Balser D. (2011). Asian Longhorned Beetle. Ohio Department of Natural Resources Division of Forestry. http://forestry.ohiodnr.gov/news/post/august-is-tree-check-month-check-for-asian-longhorned-beetle.

[8] Morewood W.D., Hoover K., Neiner P., McNeil J., Sellmer J.C. (2004). Host tree resistance against the polyphagous wood-boring beetle *Anoplophora glabripennis*. Entomol. Exp. Appl..

[9] Zhang F., Jin Y., Chen H., Wu X. (2008). Selectivity mechanism of *Anoplophora glabripennis* on four different species of maples. Front. Biol. China.

[10] USDA-APHIS. https://www.aphis.usda.gov/aphis/ourfocus/planthealth/plant-pest-and-disease-programs/pests-and-diseases/asian-longhorned-beetle/asian-longhorned-beetle.

[11] (2006). GAO Invasive Forest Pests: Lessons Learned from Three Recent Infestations May Aid in Managing Future Efforts: Report to the Chairman, Committee on Resources, House of Representatives. https://www.gao.gov/assets/250/249776.pdf.

[12] Nowak D.J., Pasek J.E., Sequeira R.A., Crane D.E., Mastro V.C. (2001). Potential effect of *Anoplophora glabripennis* (Coleoptera: Cerambycidae) on urban trees in the United States. J. Econ. Entomol..

[13] Zhang A., Oliver J.E., Aldrich J.R., Wang B., Mastro V.C. (2002). Stimulatory beetle volatiles for the Asian longhorned beetle, *Anoplophora glabripennis* (Motschulsky). Z. Naturforsch..

[14] Zhang A., Oliver J.E., Aldrich J.R. (2001). Aggregation Pheromone for the Asian Longhorned Beetle, Anoplophora glabripennis (Coleoptera: Cerambycidae). U.S. Patent.

[15] Nehme M.E., Keena M.A., Zhang A., Baker T.C., Hoover K. (2009). Attraction of *Anoplophora glabripennis* to male-produced pheromone and plant volatiles. Environ. Entomol..

[16] Crook D.J., Lance D.R., Mastro V.C. (2014). Identification of a potential third component of the male-produced pheromone of *Anoplophora glabripennis* and its effect on behavior. J. Chem. Ecol..

[17] Nehme M., Keena M.A., Zhang A., Baker T.C., Xu Z., Hoover K. (2010). Evaluating the use of male-produced pheromone components and plant volatiles in two trap designs to monitor *Anoplophora glabripennis*. Environ. Entomol..

[18] Meng P.S., Trotter R.T., Keena M.A., Baker T.C., Yan S., Schwartzberg E.G., Hoover K. (2014). Effects of pheromone and plant volatile release rates on trapping *Anoplophora glabripennis* (Coleoptera: Cerambycidae) in China. Environ. Entomol..

[19] Xu T., Hansen L., Dong C.H., Hao D., Zhang L., Teale S.A. (2020). Identification of a female-produced pheromone in a destructive invasive species: Asian longhorn Beetle, *Anoplophora glabripennis*. J. Pest Sci..

[20] Hanks L.M., Millar J.G. (2013). Field bioassays of cerambycid pheromones reveal widespread parsimony of pheromone structures, enhancement by host plant volatiles, and antagonism by components from heterospecifics. Chemoecology.

[21] Meier L.R., Zou Y., Millar J.G., Mongold-Diers J.A., Hanks L.M. (2016). Synergism between enantiomers creates species-specific pheromone blends and minimizes cross-attraction for two species of cerambycid beetles. J. Chem. Ecol..

[22] Crook D.J., Khirimian A., Francese J.A., Fraser I., Poland T.M., Sawyer A.J., Mastro V.C. (2008). Development of a host-based semiochemical lure for trapping emerald ash borer, *Agrilus planipennis* (Coleoptera: Buprestidae). Environ. Entomol..

[23] Kendra P.E., Niogret J., Montgomery W.S., Deyrup M.A., Epsky N.D. (2015). Cubeb oil lures: Terpenoid emissions, trapping efficacy, and longevity for attraction of redbay ambrosia beetle (Coleoptera: Curculionidae: Scolytinae). J. Econ. Entomol..

[24] Ginzel M.D., Hanks L.M. (2005). Role of host plant volatiles in mate location for three species of longhorned beetles. J. Chem. Ecol..

[25] Zhang A., Oliver J.E., Chauhan K., Zhao B., Xia L., Xu Z. (2003). Evidence for contact sex recognition pheromone of the Asian longhorned beetle, *Anoplophora glabripennis* (Coleoptera: Cerambycidae). Naturwissenschaften.

[26] Attygalle A.B., Zlatkis A., Middleditch B.S. (1989). Derivitization with 1,1-dimethylhydrazine for identification of carbonyl compounds resulting from ozonolysis. J. Chromatogr..

[27] McDaniel C.A., Howard R.W. (1985). Mass spectral determination of aldehydes, ketone, and carboxylic acids using 1,1-dimentylhydrazine. J. Chem. Ecol..

[28] Corey E.J., Suggs J.W. (1975). Pyridinium chlorochromate. An efficient reagent for oxidation of primary and secondary alcohols to carbonyl compounds. Tetrahedron Lett..

[29] Crook D.J., Higgins R.A., Ramaswamy S.B. (2003). Antennal morphology of the soybean stemborer *Dectes texanus texanus* LeConte (Coleoptera: Cerambycidae). J. Kans. Entomol. Soc..

[30] Lacey E.S., Ginzel M.D., Millar J.G., Hanks L.M. (2004). Male-produced aggregation pheromone of the cerambycid beetle *Neoclytus acuminatus acuminatus*. J. Chem. Ecol..

[31] R Core Team (2019). R: A Language and Environment for Statistical Computing.

[32] Heinze G., Schemper M. (2002). A solution to the problem of separation in logistic regression. Stat. Med..

[33] Kosmidis I. (2018). brglm2: Bias Reduction in Generalized Linear Models; R Package Version 0.1.8.2. https://cran.r-project.org/package=brglm2/brglm2.pdf.

[34] Lenth R. (2024). Emmeans: Estimated Marginal Means, Aka Least-Squares Means, R Package Version 1.10.0. https://cran.r-project.org/web/packages/emmeans/emmeans.pdf.

[35] Collignon R.M., Swift I.P., Zou Y., McElfresh J.S., Hanks L.M., Millar J.G. (2016). The influence of host plant volatiles on the attraction of longhorn beetles to pheromones. J. Chem. Ecol..

[36] Allison J.D., McKenna J.L., Millar J.G., McElfresh J.S., Mitchell R.F., Hanks L.H. (2012). Response of the woodborers *Monochamus carolinensis* and *Monochamus titillator* to known cerambycid pheromones in the presence and absence of the host plant volatile α-pinene. Environ. Entomol..

[37] Fierke M.K., Skabeikis D.D., Millar J.G., Teale S.A., McElfresh J.S., Hanks L.M. (2012). Identification of a male-produced pheromone for *Monochamus scutellatus* and an attractant for the congener *Monochamus notatus* (Coleoptera: Cerambycidae). J. Econ. Entomol..

[38] Ryall K., Silk P., Webster R.P., Gutowski J.M., Meng Q., Li Y., Gao W., Fidgen J., Kimoto T., Scarr T. (2014). Further evidence that monochamol is attractive to Monochamus (Coleoptera: Carambycidae) species, with attraction synergized by host plant volatiles and bark beetle (Coleoptera: Curculionidae) pheromones. Can. Entomol..

[39] Teale S.A., Wickham J.D., Zhang F., Su J., Chen Y., Xiao W., Hanks L.M., Millar J.G. (2011). A male-produced aggregation pheromone of Monochamus alternatus (Coleoptera: Cerambycidae), a major vector of pine wood nematode. J. Econ. Entomol..

[40] Nehme M.E., Trotter R.T., Keena M.A., McFarland C., Coop J., Hull-Sanders H.M., Meng P., De Moraes C.M., Mescher M.C., Hoover K. (2014). Development and evaluation of a trapping system for *Anoplophora glabripennis* (Coleoptera: Cerambycidae) in the United States. Environ. Entomol..

[41] Wickham J.D. (2009). Semiochemicals of the Asian Longhorn Beetle, *Anoplophora glabripennis* (Motschulsky) Coleoptera: Cerambycidae). Ph.D. Thesis.

[42] Tooker J.F., Koenig W.A., Hanks L.M. (2002). Altered host plant volatiles are proxies for sex pheromones in the gall wasp Antistrophus rufus. Proc. Natl. Acad. Sci. USA.

[43] Smith M., Tobin P., Wu J., He W., Xu X., Gries G., Gries R., Borden J., Turgeon J., de Groot P. (2007). Detection and monitoring of the Asian longhorned beetle: Update on sentinal trees, attract and kill, and artificial lure studies. Proceedings of the Emerald Ash Borer Research Technology Development Meeting.

[44] Smith M., Tobin P., Wu J., He W., Xu X., Gries G., Gries R., Borden J., Turgeon J., de Groot P. (2008). Behavioral ecology of host selection in the Asian longhorned beetle: Implications for surveying, detecting, and monitoring adult beetles. Proceedings of the 18th U.S. Department of Agriculture Interagency Research Forum on Gypsy Moth and Other Invasive Species.

[45] Yasui H., Akino T., Fukaya M., Wakamura S., Ono H. (2008). Sesquiterpene hydrocarbons: Kairomones with a releaser effect in the sexual communication of the white-spotted longicorn beetle, *Anoplophora malasiaca* (Thomson) (Coleoptera: Cerambycidae). Chemoecology.

[46] Yasui H. (2009). Chemical communication in mate location and recognition in the white-spotted longicorn beetle, *Anoplophora malasiaca* (Coleoptera: Cerambycidae). Appl. Entomol. Zool..

[47] Wei J., Zhou Q., Hall L., Myrick A., Hoover K., Shields K., Baker T.C. (2018). Olfactory sensory neurons of the Asian longhorned beetle *Anoplophora glabripennis*, specifically responsive to its two aggregation-sex pheromone components. J. Chem. Ecol..

[48] Blomquist G.J., Ginzel M.D. (2021). Chemical ecology, biochemistry, and molecular biology of insect hydrocarbons. Annu. Rev. Entomol..

[49] Baeckstrom P., Bjorkling F., Hogberg H.E., Norin T. (1984). Cross-coupling of vinyl cuprates and allylic halides and synthesis of the Comstock mealybug pheromone via photo-oxidation of 2,6-dimethyl-2, 5-heptadiene. Acta Chem. Scand. B.

[50] Faal H., Canlas I.J., Cossé A., Jones T.H., Carrillo D., Cooperband M.F. (2023). Investigating photo-degradation as a potential pheromone production pathway in spotted lanternfly, Lycorma delicatula. Insects.

[51] Bartelt R.J., Cossé A.A., Petroski R.J., Weaver D.K. (2002). Cuticular hydrocarbons and novel alkenediol diacetates from wheat stem sawfly (Cephus cinctus): Natural oxidation to pheromone components. J. Chem. Ecol..

[52] Faal H., Silk P.J., LeClair G., Teale S.A. (2022). Biologically active cuticular compounds of female Sirex noctilio. Entomol. Exp. Appl..

[53] Staples J.K., Bartelt R.J., Cossé A.A., Whitman D.W. (2009). Sex pheromone of the pine false webworm Acantholyda erythrocephala. J. Chem. Ecol..

[54] Lebreton S., Borrero-Echeverry F., Gonzalez F., Solum M., Wallin E.A., Hedenström E., Hansson B.S., Gustavsson A.L., Bengtsson M., Birgersson G.A. (2017). Drosophila female pheromone elicits species-specific long-range attraction via an olfactory channel with dual specificity for sex and food. BMC Biol..

[55] Cossé A.A., Bartelt R.J., Weaver D.K., Zilkowski B.W. (2002). Pheromone components of the wheat stem sawfly: Identification, electrophysiology, and field bioassay. J. Chem. Ecol..

[56] Bartelt R.J., Jones R.L. (1983). (Z)-10-nonadecenal: A pheromonally active air oxidation product of (Z,Z)-9, 19 dienes in yellowheaded spruce sawfly. J. Chem. Ecol..

[57] Swedenborg P.D., Jones R.L. (1992). (Z)-4-tridecenal, a pheromonally active air oxidation product from a series of (Z,Z)-9-13 dienes in *Macrocentrus grandii* Goidanich (Hymenoptera: Braconidae). J. Chem. Ecol..

[58] Hoover K., Keena M., Nehme M., Wang S., Meng P., Zhang A. (2014). Sex-specific trail pheromone mediates complex mate finding behavior in *Anoplophora glabripennis*. J. Chem. Ecol..

[59] Lacey E.S., Ray A.M., Hanks L.M. (2007). Calling behavior of the cerambycid beetle *Neoclytus acuminatus acuminatus* (F.). J. Insect Behav..

[60] Graves F., Baker T.C., Zhang A., Keena M., Hoover K. (2016). Sensory aspects of trail-following behaviors in the Asian longhorned beetle *Anoplophora glabripennis*. J. Insect Behav..

[61] Carlson D.A., Mayer M.S., Silhacek D.L., James J.D., Beroza M., Bierl B.A. (1971). Sex attractant pheromone of the house fly: Isolation, identification and synthesis. Science.

[62] Lima I., Tadeo E., Remedios-Mendoza M., Martinez-Hernández M., Ruiz-Montiel C. (2023). Evidence of a pheromone involved in the behaviour of *Drosophila suzukii* Matsumura (Diptera: Drosophilidae). J. Appl. Entomol..

[63] Frankel E.N. (1998). Lipid Oxidation.

